# Peer victimization in single‐grade and multigrade classrooms

**DOI:** 10.1002/ab.21851

**Published:** 2019-06-26

**Authors:** J. Ashwin Rambaran, Marijtje A. J. van Duijn, Jan Kornelis Dijkstra, René Veenstra

**Affiliations:** ^1^ Department of Sociology and Interuniversity Center for Social Science Theory and Methodology (ICS) University of Groningen The Netherlands

**Keywords:** classroom context, dominance, evolutionary, peer victimization, social networks

## Abstract

Although peer victimization mainly takes place within classrooms, little is known about the impact of the classroom context. To this end, we examined whether single‐grade and multigrade classrooms (referring to classrooms with one and two grades in the same room) differ in victim–bully relationships in a sample of elementary school children (646 students; age 8–12 years; 50% boys). The occurrence of victim–bully relationships was similar in single‐grade and multigrade classrooms formed for administrative reasons, but lower in multigrade classrooms formed for pedagogical reasons. Social network analyses did not provide evidence that peer victimization depended on age differences between children in any of the three classroom contexts. Moreover, in administrative multigrade classrooms, cross‐grade victim–bully relationships were less likely than same‐grade victim–bully relationships. The findings did not indicate that children in administrative multigrade classrooms are better or worse off in terms of victim–bully relationships than are children in single‐grade classrooms.

## 1 INTRODUCTION

Peer victimization is a pervasive and reoccurring societal problem: it is repeatedly shown that it mainly harms those who are socially the most vulnerable in school (Farris & Felmlee, 2011, 2014) and comes with a high cost as targets suffer from social, emotional, and physical health problems (for an overview, see Rivara & Le Menestrel, 2016). Increasingly, researchers have come to recognize the important role of classroom characteristics in peer victimization (Juvonen & Graham, 2014), and realize that peer victimization depends on the characteristics of the perpetrator, the target, and the social context (Salmivalli, Lagerspetz, Björkqvist, Österman, & Kaukiainen, 1996; Volk, Camilleri, Dane, & Marini, 2012).

The classroom provides both a context and a frame of reference in which social dominance hierarchies are established based on social interactions between children (Farmer, Lines, & Hamm, 2011). These interactions typically take place within same‐age peer groups as most school children across the world are traditionally organized in single‐grade classrooms (Mulryan‐Kyne, 2005). However, interactions can also take place within mixed‐age peer groups as schools increasingly combine different grades within one group, so‐called multigrade classrooms (Mulryan‐Kyne, 2007; Veenman, 1995). Thus far, research into peer victimization has neglected age‐mixing as a topic of study (Ellis et al., 2012). This is surprising because age differences are a natural source of power imbalance, which is one important feature of bullying (Salmivalli, 2010). The multigrade classroom provides the opportunity to investigate whether peer victimization depends on age differences between classmates as it provides a context where age differences between the children are larger than in single‐grade classrooms.

Two perspectives may explain peer victimization. Following a status framework (Rodkin, Espelage, & Hanish, 2015), it can be argued that because of a power imbalance, younger children are more likely to be perpetrators or targets in classrooms with larger age differences (Chaux & Castellanos, 2015), such as in multigrade classrooms. Alternatively, from an evolutionary perspective (Ellis et al., 2012), it can be argued that multigrade classrooms encourage prosocial behavior in children, resulting in a lower risk of peer victimization for younger children by older children, particularly in multigrade classrooms formed for pedagogical reasons rather than administrative reasons. In this study, we distinguish between the two classroom types because pedagogical multigrade classrooms aim to stimulate prosocial relations among children by encouraging the provision of help across grades within the same classroom (Gray, 2011; Lillard & Else‐Quest, 2006), whereas administrative multigrade classrooms do not have such an explicit goal.

Currently, it is unclear whether multigrade classrooms in comparison to single‐grade classrooms are either a risk or protective factor for peer victimization in childhood. We aimed to explore the two competing perspectives by examining victim–bully relationships in single‐grade and multigrade classrooms in a sample of elementary school children. Investigating classroom differences is an important direction in bullying research as a better understanding of the role of age differences in the classroom may help to advance prevention or intervention policies.

### 1.1 A status framework to understand peer victimization

One perspective toward explaining peer victimization is the status framework (Rodkin et al., 2015), which posits that bullying is targeted peer aggression within the context of a relationship of power and abuse. In such relationships of power imbalance, perpetrators are aggressors repeatedly targeting socially vulnerable individuals to maintain or gain a high social status (Salmivalli, 2010; Veenstra, Lindenberg, Munniksma, & Dijkstra, 2010). Targets of peer bullying thus suffer from their disadvantaged and isolated position in the group as they form easy targets because of their low status (Farmer et al., 2011). In this perspective, bullying perpetration is instrumental to acquire social resources and to maintain dominance over marginalized peers (Rodkin et al., 2015), and to demonstrate self‐esteem and social skills (Guerra, Williams, & Sadek, 2011). This further enhances the perpetrators’ positions in the group and weakens the positions of targets.

Age forms an important aspect through which social dominance hierarchies can be determined in classrooms (Ellis et al., 2012). As children grow older, they become socially, cognitively, and physically more developed (Piaget, 1953). Older children thus have a clear advantage over younger children, to obtain dominant positions in the group. They may use this advantage by targeting younger children to demonstrate social dominance. Younger children compared with older children are more vulnerable for peer victimization (Barker et al., 2008; Chaux, Molano, & Podlesky, 2009; Rivers & Smith, 1994; Scheithauer, Hayer, Petermann, & Jugert, 2006) as they are more likely to have a weaker social position in the peer group (Salmivalli, 2010). This suggests that younger children are an easy target for older children.

It makes sense that an effect of power imbalance through age would be more salient in a context with a large age‐range. In contrast to single‐grade classrooms where an age difference between the children of 1 year is usually the maximum to be expected, children in multigrade classrooms differ up to the number of grades combined in the classroom. Considering that students spend most of their time in school within the same classroom, this means that in single‐grade classrooms, social interactions mostly take place in same‐age groups, whereas in multigrade classrooms they also take place among children who clearly differ in age. Researchers have argued that as a consequence of age‐mixing, multigrade classrooms may produce peer hierarchies based on students’ age and thus, putting younger children at risk for victimization (Kolbert & Crothers, 2003). Hence, in multigrade classrooms older children may target younger children as a means to demonstrate dominance.

Following this line of reasoning, we expected that power imbalance would be more related to age differences in multigrade classrooms compared with single‐grade classrooms, and, therefore, in multigrade classrooms we expected to find higher degrees of victimization (H1a), and higher risk of peer victimization for younger children targeted by older children (H2a). In multigrade classrooms, we can separate the age difference in a grade effect and a relative age difference, whereas in single‐grade classrooms only the (relative) age difference can be examined.

### 1.2 An evolutionary model to understand peer victimization

The evolutionary model of risky child/adolescent behavior provides an alternative perspective toward explaining peer victimization in classrooms (Ellis et al., 2012). This model posits that mixed‐age settings, rather than age‐segregated school and peer environments, are the natural context for child development. The presence of both older and younger children in mixed‐age settings provides a natural hierarchy based on age. In this context, both older and younger children settle with their position in the social group, which decreases the tendency to compete for dominance and status by demonstrating aggressiveness. Evolutionary psychologists argue that older children can serve as positive role models, and that the positive association between status and prosocial behavior reduces the need to gain status through antisocial means (Ellis et al., 2012). The research findings show that when older children are assigned to younger children as caregivers, buddies, or playmates, they tend to behave less aggressive and more prosocial toward younger children and same‐age peers in other contexts as well (Ember, 1973; Gray, 2011). In addition, the findings suggest that children in mixed‐age school settings interact socially across wide age ranges; older children and younger children associate with each other, and become friends with each other (Miller, 1990; Pratt, 1986). In sum, this study suggests that the presence of younger children in mixed‐age settings reduces aggression and promotes nurturance and compassion in children (Gray, 2011). In contrast, age‐segregated school and peer environments, such as single‐grade classrooms, have been argued to evoke aggression and conflict in children, and, in such a classroom context, children may actively search for dominance (Ellis et al., 2012).

There are two reasons why multigrade classrooms are formed within schools (Veenman, 1995). First, schools deliberately form such classrooms for didactic and pedagogical purposes, for instance to enhance the classroom climate. An example of such schools is Montessori schools, which are characterized by a special set of educational materials, freedom as a student to choose own activities, collaboration between students, and individual and small group instruction in both academic and social skills (Lillard & Else‐Quest, 2006). Second, schools tend to form such classrooms for administrative reasons, for example, when dealing with low enrollment and uneven classroom sizes. It is important to make a distinction between these two forms of multigrade classrooms as the basis for their formation may yield different outcomes with regard to the victim–bully relationships. Particularly pedagogical multigrade classrooms are argued and shown to have more positive outcomes because promoting prosocial behavior between the older and younger children in such classrooms is part of the school's educational philosophy (Lillard & Else‐Quest, 2006; Moller, Forbes‐Jones, & Hightower, 2008). By contrast, teachers in administrative multigrade classrooms were generally found to teach the grades separately (Mulryan‐Kyne, 2007; Veenman, 1995; but see Mason & Burns, 1996), which decreases opportunities for prosocial behavior between older and younger children as such multigrade classrooms emphasize individualized work and do not necessarily encourage between‐grade interactions (Juvonen, 2018).

Following this line of reasoning, we formulated two additional hypotheses. In pedagogical multigrade classrooms, we expected to find lower degrees of victimization (H1b) and lower risk of victimization for children targeted by older children (H2b).

### 1.3 The present study

We investigate victim–bully relationships in single‐grade and multigrade classrooms. We addressed two main questions: (a) Do multigrade classrooms show different degrees of victimization compared to single‐grade classrooms? (b) To what extent does peer victimization depend on age differences between children, and if so, is this different for single‐grade and multigrade classrooms? We focused on middle to late childhood, because in that period social dominance hierarchies are established through school bullying (Kolbert & Crothers, 2003). We controlled for sex, because boys are often more dominant and aggressive toward peers than girls (Cook, Williams, Guerra, Kim, & Sadek, 2010).

## 2 METHOD

### 2.1 Sample

Classrooms were drawn from the second wave of the KiVa study at the start of the school year (in October 2012). KiVa is a program aimed to reduce school bullying among children from grades 3–6 in elementary education (8–12 years) in the Netherlands (Huitsing et al., 2019; Kaufman, Kretschmer, Huitsing, & Veenstra, 2018). The 99 participating schools (66 intervention and 33 control schools) contained 25 schools with only single‐grade classrooms, 39 schools with only administrative multigrade classrooms, 14 schools with only pedagogical multigrade classrooms, and 21 schools with both single‐grade and administrative multigrade classrooms. Only control schools were selected for the analysis to avoid that differences between classrooms were a result of the intervention. Next, we selected schools that had either only single‐grade classrooms 3–6 or only multigrade classrooms containing two grades (e.g., 3–4 or 5–6) excluding the 19 schools with other combinations.

For the remaining 14 schools, containing 38 classrooms, we applied an additional set of selection criteria for classroom's eligibility for social network analysis: first, classroom size should be larger than 15; and second, the combinations for sex (boy–boy, girl–girl, boy–girl, and girl–boy) and grade (low–low, high–high, low–high, and high–low) should contain victim–bully relationships for the reference category. The latter criterion was needed for estimating the sex and grade effects comparable across classrooms. We chose boy–boy (meaning boys nominating other boys as their perpetrator) and low–low (meaning lower grade classmates nominating other lower grade classmates as their perpetrator) as reference. The two selection criteria resulted in dropping 12 classrooms (see for details Appendix A). The final sample consisted of 26 classrooms with 646 students (see Appendix A), of which 11 single‐grade classrooms (*n* = 274), nine administrative multigrade classrooms (*n* = 216), and six pedagogical multigrade classrooms (*n* = 156). Information about the school's pedagogical background was provided by the school office, and was used to categorize the schools into the three categories.

Appendix B shows the differences between the reduced sample (the 38 classrooms) and the final network sample (the 26 classrooms). The two samples were comparable in classroom size and percentage of boys, but small differences were observed for average nominations sent for bullying perpetration per student and for the distribution of these nominations. Average degree among pedagogical multigrade classrooms was slightly higher in the network sample compared to the reduced sample (0.5 versus 0.4), whereas the distribution of degrees was slightly more skewed among administrative multigrade classrooms (see Appendix B).

In our subsample of 646 students, 324 (50.2%) students were boys and 322 (49.8%) students were girls. The average age of the sample was 10.2 years (*SD* = 1.2). Most students (*n* = 529; 81.9%) were native Dutch; 16.7% were non‐Dutch, 1.4% was missing because nine children provided insufficient information about their parent's ethnic background.

### 2.2 Procedure

Students filled in an internet‐based questionnaire in their classroom during regular school hours. The process was administered by the teachers, who were present to answer questions and to assist the students when necessary. Before the data collection, teachers were given detailed instructions concerning the administration of questionnaires to students. During the data collection, support was available through phone and email.

At the beginning of the questionnaire, students received information about the goal of the study, and how to fill in the questionnaire. They were told not to talk to each other or to discuss their answers when they filled out the questionnaire or afterward to ensure each other's privacy. It was explained to students that their answers would remain confidential. The teachers ensured that students who could not complete the questionnaire on the day of the data collection participated at another day within a month.

Before the first wave (the pre‐assessment) of the KiVa study (and for students who were new in school, after the first wave), schools sent information letters to students’ parents. A passive consent procedure allowed students or parents to opt out of students’ participation. At the start of data collection (2012), universities in the Netherlands did not require IRB permission for this type of research. All procedures performed were in accordance with the 1964 Helsinki Declaration and its later amendments or comparable ethical standards. A few students did not want to participate; also a few parents objected to their child's participation. Accordingly, for the whole sample at the second wave of the KiVa study participation rate was high (96%).

### 2.3 Measures

#### 2.3.1 Dependent variable

Peer victimization (bullying perpetration) was measured with network nominations following a two‐stage procedure. Students were first asked to indicate how often they were victimized in general in the previous months (since the summer break), according to Olweus’ (1996) self‐reported bully/victim items, and, to indicate this for specific form(s) of victimization; physical harm (e.g., kicked), verbal harm (e.g., name‐calling), relational harm (e.g., gossiping), and cyber victimization. Answers were given on a five‐point scale: “Not at all” (1), “Once or twice” (2), “Two or three times a month” (3), “Once a week” (4), and “Several times a week” (5).

When participants indicated that they were victimized by classmates at least “Once or twice” (score 2) on any item, they were presented with a roster showing the names of all classmates, and asked by whom of their classmates they were victimized (referring to “Who starts bullying you?”). Perpetration nominations were measured as either present (1) or absent (0). Students who indicated not being victimized by classmates did not fill out the nomination question. Their “answers” were considered as “structural missing” (no outgoing nomination possible). This means that victims were the only one “allowed” to send a perpetration nomination to classmates, but that everyone could receive a perpetration nomination. Victim–bully networks were obtained based on all perpetration nominations in a particular classroom (from the target's perspective).

#### 2.3.2 Independent variables

We included *sex* (1 = boy) as a control. Students’ *grade* was obtained from the school's office. The multigrade classrooms in our study included two grades in each classroom (3–4 or 5–6); students were categorized as belonging either to the lower grade or to the higher grade in their classroom. Students’ birth dates were also obtained from the school's office. Age was derived from birth date and measured in number of months. We computed *relative age* by subtracting the median age in the classroom (calculated among all students in the classroom) from students’ own age. Relative age in multigrade classrooms was obtained by subtracting 12 months from the higher grade students’ age, to compensate for a 1‐year age difference between the two grades. This way, children's age in single‐grade and multigrade classrooms were made comparable to separate grade differences from (relative) age differences.

### 2.4 Analytic strategy

The investigation of the victim–bully relationships in single‐grade and multigrade classrooms in elementary school was done with two analyses. We tested our first set of hypotheses (H1a and H1b) using nonparametric analysis using Kruskal–Wallis *H* tests, and our second set of hypotheses (H2a and H2b) using cross‐sectional social network analysis using ERGMs (Exponential Random Graph Models; Lusher, Koskinen, & Robins, 2013). The Kruskal–Wallis test is used to test differences in the median scores of tie percentage at the classroom level (average in‐degrees) across the three classroom types (single‐grade, administrative multigrade, and pedagogical multigrade). ERGMs can be used to analyze cross‐sectional social network structures (Robins, 2015), and have been used before to examine victim–bully relationships (Huitsing et al., 2012). This choice allows us to simultaneously investigate individual and dyadic age and grade effects while explicitly taking into account the dependencies in the victim–bully network.

Cross‐sectional social network analysis was done in PNet (Wang, Robins, & Pattison, 2009), a software program for the statistical analysis of social network data using ERGMs. The effects were first analyzed per classroom network, and were then meta‐analyzed in R with classroom type as “explanatory variable” (referring to a mixed‐effects model in metafor, see Viechtbauer, 2010). Each classroom network was estimated with the same model specification. For some classroom networks, however, some parameters were left out, because the accompanying statistics (network configurations) were not there. The usual criteria for convergence (absolute value of *t* statistics below 0.10 for all parameters; see Wang et al., 2009) was obtained for all classroom networks. Appendix C provides Goodness of Fit (GoF) statistics. As shown, classroom networks adhered to the usual criteria for acceptable GoF statistics (absolute value of *t* statistics below 2). In four classrooms (7, 8, 12, and 23), the fit was not optimal for the reciprocal age‐related effects. Because we already included nonreciprocal age effects, including additional age effects resulted in non‐convergence.

### 2.5 Model specification and effect interpretation

To adequately capture important features of the victim–bully networks, we followed previous research in choosing the structural parameters in the exponential random graph models (Huitsing & Veenstra, 2012; Huitsing et al., 2012), using alternating or geometrically weighted versions of the structural statistics. *Density* indicates the general occurrence of peer victimization ties, comparable with the intercept or grand mean in (generalized) linear models. The *isolates* parameter indicates the occurrence of a network configuration where a child does not nominate others as their perpetrators or receive a perpetration nomination from others (noninvolvement), whereas the *sinks* parameter indicates the occurrence of a configuration where a child receives a perpetration nomination, but does not nominate anyone (referring to perpetrators who are not targets themselves). The *multiple two‐paths* parameter reflects a perpetrator‐target, referring to a child receiving a perpetration nomination and nominating a perpetrator. The *in‐ties spread* parameter represents variability in receiving nominations as a perpetrator, whereas the *shared in‐ties* parameter represents variability in sending perpetration nominations by some children attracting more perpetration nominations than others. If a structural parameter is significant, this indicates that the structural configuration occurs more frequently than if perpetration nominations would be formed at random. We included sex as a covariate by specifying dyadic covariates indicating the sex combinations (taking the boy–boy dyad as the reference category).

To test the hypotheses H2a and H2b, individual and dyadic age and grade effects were included in the models. Thus, the effect of age difference is separated in three components that are comparable over the three classroom types, distinguishing differences due to relative age or grade, while controlling for a “main” or general age effect by a receiver effect of the relative age of the child. Note that no grade difference was defined for the single grade classrooms.

## 3 RESULTS

### 3.1 Descriptive findings

Table 1 presents the summarized descriptive findings of the three types of classrooms. Appendix D provides information per classroom. Peer victimization (bullying perpetration) was, on average, almost two times as low in pedagogical multigrade classrooms (av. degree = 0.5) compared with both single‐grade classrooms and administrative multigrade classrooms, which were comparable in degrees of peer victimization (av. degree = 0.9).

**Table 1:** Overview of the single‐grade and multigrade classrooms included in this study

		Regular single‐grade	Administrative multigrade	Pedagogical multigrade
**Sample description** (classes)				
No. of students		274 (11)	216 (9)	156 (6)
Single‐grade	Multigrade			
Grade 3	Grade 3–4	74 (3)	88 (4)	78 (3)
Grade 4		84 (3)		
Grade 5	Grade 5–6	66 (3)	128 (5)	78 (3)
Grade 6		50 (2)		
Av. classroom size (min–max)		25 (19–32)	26 (18–30)	26 (22–29)
Av. proportion boys (min–max)		0.48 (0.32–0.60)	0.52 (0.39–0.70)	0.51 (0.42–0.61)
**Age in months** (median, min–max)				
Single‐grade	Multigrade			
Av. grade 3	Grade 3–4	104 (94–120)	110 (93–135)	108 (95–124)
Av. grade 4		117 (104–131)		
Av. grade 5	Grade 5–6	129 (119–145)	135 (114–156)	134 (119–153)
Av. grade 6		140 (132–156)		
**Network density** (min–max)^a^				
Av. ties		21 (5–39)	21 (11–38)	12 (5–24)
Av. degree		0.9 (0.2–2.0)	0.9 (0.4–1.7)	0.5 (0.2–1.1)
Av. density		0.04 (0.01–0.10)	0.04 (0.01–0.09)	0.02 (0.01–0.05)
**Network density for sex**(min–max)^b^				
Av. density boy–boy		0.06 (0.01–0.11)	0.06 (0.01–0.13)	0.03 (0.01–0.08)
Av. density boy–girl		0.01 (0.00–0.07)	0.02 (0.00–0.08)	0.01 (0.00–0.02)
Av. density girl–girl		0.02 (0.00–0.14)	0.02 (0.00–0.05)	0.01 (0.00–0.03)
Av. density girl–boy		0.07 (0.00–0.27)	0.05 (0.01–0.16)	0.04 (0.01–0.11)
**Network density for grade**(min–max)^b^				
Av. density low–low		–	0.04 (0.00–0.12)	0.03 (0.00–0.09)
Av. density low–high		–	0.03 (0.00–0.07)	0.02 (0.00–0.05)
Av. density high–high		–	0.06 (0.01–0.10)	0.02 (0.00–0.06)
Av. density high–low		–	0.04 (0.00–0.11)	0.02 (0.00–0.03)

^a^ = Density is the proportion of observed ties present in the network in relation to the total amount of possible ties available in the network.

^b^ = Density for sex or grade is based on the proportion of ties present in each group in relation to the total amount of possible ties available in each group.

Table 1 also shows on average higher bullying involvement with boys: they nominated mainly each other as perpetrators, and were also nominated as perpetrators by girls. In multigrade classrooms, bullying perpetration nominations occurred mainly among same‐grade students: most students indicated that they were being targeted by a peer in their own grade (see av. densities for the low–low group and the high–high group in Table 1).

Appendix D shows the relative age differences between targets’ perpetrators and targets’ nonperpetrators, showing no clear pattern as children were targeted approximately equally often by older and younger classmates, except for the pedagogical 5–6 grades, where targets more often had older perpetrators.

The Kruskal–Wallis test shows no statistically significant differences in the median scores of tie percentage at the classroom level (average in‐degrees) between the three classroom types (single‐grade, administrative multigrade, and pedagogical multigrade). This means that there was no evidence that the three classroom types differ in victimization (*χ* ^2^ (2, *n* = 26) = 4.5, *p* = .11), with a median rank of 14 for single‐grade classrooms, 17 for administrative multigrade classrooms, and 5.8 for pedagogical multigrade classrooms.

### 3.2 ERGM findings

Table 2 presents the summary of the ERGMs. Appendix E shows the results per classroom. We present our findings leaving out two classrooms that were identified as outliers (see Appendix E). Removing these classrooms from the meta‐analysis, led to more reliable results (referring to less variation across classrooms and results were not more “favorable;” compare Table 2 with Appendix F). The first column in Table 2 shows the mean estimates across administrative multigrade classrooms (as reference), the two other columns show the degree to which regular single‐grade and pedagogical multigrade classrooms deviate from this.

**Table 2:** ERGM meta‐analysis results for victimization networks in single‐grade and multigrade classrooms

			Intercept (administrative multigrade)		Intercept + regular single‐grade		Intercept + pedagogical multigrade	
Parameter	Illustration	*n*	Est.	(*SE*)	Est.	(*SE*)	Est.	(*SE*)
Network effects								
Density (Arc)	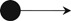	24	−3.16***	(0.66)^a^	0.28	(0.89)	−1.06	(1.02)
Sinks (sink)	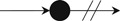	24	1.31**	(0.49)	−0.10	(0.66)	0.05	(0.76)
Isolates (isolates)	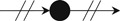	24	1.51**	(0.48)	0.31	(0.65)	−0.14	(0.73)
In‐ties spread (AinS)	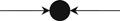	21	0.63+	(0.36)	−0.14	(0.51)	−0.12	(0.62)
Multiple two‐paths (A2P‐T)	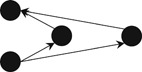	18	−0.02	(0.18)	−0.06	(0.24)	0.17	(0.32)
Shared in‐ties (A2P‐D)	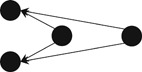	19	0.11	(0.16)	0.03	(0.21)	−0.003	(0.29)
Sex effects								
Boy–boy (ref.cat.)	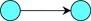	24	–	–	–	–	–	–
Girl–girl	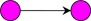	18	0.16	(0.41)	−0.42	(0.57)	−0.51	(0.66)
Girl–boy	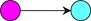	23	0.16	(0.28)	0.25	(0.38)	0.53	(0.46)
Boy–girl	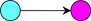	15	−0.27	(0.38)	−0.21	(0.57)	−0.45	(0.63)
Grade effects								
Low–low (ref.cat.)	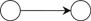	13	–	–	–	–	–	–
High–high	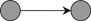	11	0.32	(0.31)	–	–	0.12	(0.53)
Low–high	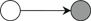	12	−0.36	(0.38)	–	–	1.37*	(0.63)
High–low	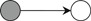	11	−0.30	(0.33)	–	–	0.25	(0.54)
Age effects								
Age‐receiver	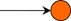	24	0.03	(0.08)	0.01	(0.11)	−0.02	(0.15)
Difference in (relative) age	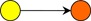	24	−0.01	(0.07)	0.03	(0.09)	0.01	(0.11)
	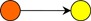							

*Notes*: + *p ≤ *.10, *p ≤ .05, **p ≤ .01, ***p ≤ .001. ^a^Significant differences between classrooms. To help interpret the effects in this table, an illustration is provided for each parameter. Victim–bully relationships are represented with directed arrows (referring to targets nominating perpetrators). Students are represented with colored nodes (sex: boys in blue, girls in pink; grade: lower grade students in white, higher grade students in gray; age: older children in orange, younger children in yellow). The parameter statistics of the network effects used in PNet are mentioned in parentheses (short names).

#### 3.2.1 Network effects

The results of the basic network effects were comparable across the three classroom types (see Table 2). Taking the degree of peer victimization among boys into account, the negative *density* effects across all three classroom types indicate the low occurrence of victim–bully relationships. The positive *isolates* effects indicate that some students were uninvolved in victim–bully relationships: they were neither targets nor perpetrators. The positive *sinks* effects show that some students, while nominating none as a perpetrator themselves, were considered as perpetrators by classmates. The *in‐ties spread* effects and *shared‐in‐ties* effects, reveal that there is no clear pattern in the occurrence of variability in receiving and sending perpetration nominations. Finally, there was no evidence for the occurrence of bully‐victims patterns. Taken together the network effects implied that all three classroom types were comparable in network structure.

#### 3.2.2 Sex effects

Taking into account all other effects, the ERGMs show on average no clear sex differences in bullying involvement across the three classroom types (see Table 2). Subtle (nonsignificant) differences in the mean estimates were observed for the *girl–boy* effects (more positive in single‐grade classrooms and pedagogical multigrade classrooms) and *girl–girl* effect (more negative in single‐grade classrooms and pedagogical multigrade classrooms). Overall, the results suggest higher involvement in bullying perpetration by boys than girls in both single‐grade classrooms and pedagogical multigrade classrooms than in administrative multigrade classrooms.

#### 3.2.3 Age and grade effects

Social network analyses showed no evidence that peer victimization depended on age differences between children in any of the three classroom types: on average, older children did not receive more perpetration nominations from younger children than vice versa (nonsignificant age‐difference effects). In addition, there was no evidence that older children received more perpetrator nominations than younger children (nonsignificant age‐receiver effects). We checked whether these nonsignificant effects were a result of low sensitivity (by using a stricter cut‐off point for peer victimization, referring to being victimized two or three times a month instead of once or two times; see, Solberg & Olweus, 2003), which was not the case (see Appendix G). We also tested for sex differences, by including an interaction term for the sex combinations, but found no significant interaction effects (see Appendix H). Note that age effects were smaller than grade effects in terms of “effect size” even when assessed on a 1‐year basis.

Administrative multigrade classrooms showed a negative *low‐high* effect indicating that students in the lower grade were bullied less by students in the higher grade. In contrast, pedagogical multigrade classrooms showed a positive effect. However, this grade dominance effect was driven only by two classrooms (pedagogical multigrade classrooms 22 and 24; see Appendix E). These findings indicate that victim–bully relationships in administrative multigrade classrooms occurred mainly among same‐grade students, whereas in pedagogical multigrade classrooms they sometimes also occurred across grades.

Taken together the observations, there was no conclusive evidence for the idea that peer victimization depended on (relative) age differences or grade differences between children in classrooms.

## 4 DISCUSSION

We aimed to address the question whether age‐differences between classmates can serve as indicators for power imbalance in bullying perpetration (Ellis et al., 2012) by examining whether single‐grade and multigrade classrooms differ in peer victimization and whether peer victimization depended on (relative) age or grade differences between children. Drawing on a status framework (Rodkin et al., 2015), in multigrade classrooms we expected to find higher peer victimization, especially for younger children targeted by older children. The competing hypotheses, derived from an evolutionary model (Ellis et al., 2012), would suggest otherwise. Peer victimization was treated as a relationship between targets and perpetrators, and therefore analyzed with social network analysis. Our analyses showed no evidence that single‐grade and multigrade classrooms differ in degrees of peer victimization nor a systematic indication that peer victimization is based on (relative) age or grade differences. Thus, providing no support for either the status‐framework or the evolutionary model.

The analysis showed instead that peer victimization in administrative multigrade classrooms occurred mostly within the same grade, and thus between children whose age differences were relatively small. An explanation is that it was relatively easy for older children to target younger children and therefore targeting someone younger as they are did not enhance their status. Peers might be more inclined to award perpetrators with status when perpetrators challenge others of equal (or higher) status in the group. Likewise, younger children in the multigrade classroom were probably not in a strong position to take on older perpetrators, and therefore sought out others within their own respective lower grade. This could also explain why we found bullying among same‐age or same‐grade peers in administrative multigrade classrooms. Another explanation is that teachers in administrative multigrade classrooms generally teach the grades separately (Mulryan‐Kyne, 2007; Veenman, 1995), and this decreased opportunities for bullying between cross‐age or cross‐grade classmates.

The evolutionary model may be relevant for pedagogical multigrade classrooms. We observed differences in degrees of peer victimization between the two types of multigrade classrooms: lower degrees of peer victimization in pedagogical multigrade classrooms compared with administrative multigrade classrooms.

Findings also revealed no clear sex differences in bullying involvement across the three classroom types, after controlling for network structure, and age/grade. This is surprising given evidence on the differences between girls’ and boys’ friendship networks during the middle to late childhood developmental period (see Rose & Rudolph, 2006), with important differences found in playmate selection, friendship formation (Maccoby, 1998), and social hierarchy development for boys and girls.

It could be that age (and additionally grade in multigrade classrooms) and sex were not sufficient to investigate the effects of power imbalance in classrooms. For instance, it may be that among the youngest children only those who were physically smaller and weaker become targets of peer victimization by classmates. Hence, physical appearance or physical strength may explain differences in children's social positions rather than their age (or grade). In support of this argument, recent research has shown that peer victimization is higher among boys who are pubertal underdeveloped or those who misperceive themselves to be underweight (Haltigan & Vaillancourt, 2018; Lee, Dale, Guy, & Wolke, 2018). Within the context of bullying another important indicator of power (imbalance) to control or harm others is peer perceived popularity (Salmivalli, 2010), or more broadly defined status differences, for instance in the form of SES (Chaux & Castellanos, 2015), majority/minority status or LGBT status (Juvonen & Graham, 2014). Clearly, more precision is necessary for the measurement of power imbalance to understand how power imbalance unfolds in victim–bully relationships.

### 4.1 Implications of the research

Our findings may have implications for school policy about classroom organization. When transitioning to a multigrade classroom organization, parents worry about their child's progress and well‐being. Previous research points in the direction that children in administrative multigrade classrooms are not better or worse off in terms of socio‐cognitive outcomes (e.g., academic achievement and social adjustment; Mulryan‐Kyne, 2005; Veenman, 1995, 1996; but see Mason & Burns, 1996). In this study, we extend previous research by showing that there was also no clear indication that multigrade classrooms increased the risk of victimization by classmates compared with single‐grade classrooms.

Our findings indicated that multigrade classrooms may be beneficial for peer victimization when they are part of the school's educational philosophy. This finding is also in line with broader theory and research on the positive effects of pedagogical multigrade classrooms (e.g., in Jenaplan or Montessori schools, see Lillard & Else‐Quest, 2006; Moller et al., 2008), which stimulate prosocial behavior among children by encouraging the provision of help across the grades within the classroom (Gray, 2011; Lillard & Else‐Quest, 2006). Although based on a few classrooms, it suggests that schools that encourage prosocial relationships among children by encouraging the provision of help across grades within the same classroom (Gray, 2011; Lillard & Else‐Quest, 2006), may have less bullying between classmates. A recent study also showed that schools engaged in practices to promote inclusiveness and equity as a social‐learning program may foster openness and acceptance of others (Rivas‐Drake, Saleem, Schaefer, Medina, & Jagers, 2019). An avenue for further research could be to examine the extent to which single‐grade and (administrative and pedagogical) multigrade classrooms operate similarly or differently for positive peer relationships, such as prosocial or helping relationships in the classroom.

### 4.2 Strengths, limitations, and directions for future research

An important strength of this study is that we investigated the role of (relative) age and grade differences between classmates in victim‐bully relationships. Assessing these associations in a sample of single‐grade and multigrade classrooms further add to our understanding of classroom differences in elementary education. By applying social network analysis we did not violate the assumption of independence of observations within classrooms.

To allow for a comparison between different classrooms, our sample was relatively small because we had to exclude many schools with multigrade classrooms with a different grade combination (referring to more than two grades in the same classroom). It is possible that our null‐findings are due to low statistical power (related to the low number of classrooms that were included). Future research should validate these findings using a larger sample, preferably with larger within‐classroom grade differences.

It should also be pointed out that schools that have different grades within the same classroom as a consequence of grade/classroom mixing tended to be unstable in terms of student classroom composition over school years. Hence, an obvious next question would be how this grade/classroom mixing affects children in terms of bullying and peer victimization (Rambaran, van Duijn, Dijkstra, & Veenstra, 2019).

We did not measure victimization by peers outside the classroom because the focus of this study was on the classroom. However, part of the victimization might occur by other peers in school or outside of school. We only measured peer victimization by asking the students “Who starts bullying you?” We had good reason for this because students who initiate the bullying are often considered as “ringleader” perpetrators (Salmivalli, 2010), and thus, they may be more powerful and influential than followers who join in the bullying.

## 5 CONCLUSION

In sum, our findings did not provide evidence that (relative) age differences or grade differences play a role in peer victimization in multigrade classrooms compared with single‐grade classrooms. Apparently, processes of power imbalance in bullying are not (or only weakly) related to students’ (relative) age differences or grade differences within the same classroom.

## Supporting information

Supporting information

## References

[ab21851-bib-0001] Barker, E. D. , Boivin, M. , Brendgen, M. , Fontaine, N. , Arseneault, L. , Vitaro, F. , Bissonnette, C. , & Tremblay, R. E. (2008). Predictive validity and early predictors of peer victimization trajectories in preschool. Archives of General Psychiatry, 65, 1185–1192. 10.1001/archpsyc.65.10.1185 18838635

[ab21851-bib-0002] Chaux, E. , & Castellanos, M. (2015). Money and age in schools: Bullying and power imbalances. Aggressive Behavior, 41, 280–293. 10.1002/ab.21558 25219327

[ab21851-bib-0003] Chaux, E. , Molano, A. , & Podlesky, P. (2009). Socio‐economic, socio‐political and socio‐emotional variables explaining school bullying: A country‐wide multilevel analysis. Aggressive Behavior, 35, 520–529. 10.1002/ab.20320 19739091

[ab21851-bib-0004] Cook, C. R. , Williams, K. R. , Guerra, N. G. , Kim, T. E. , & Sadek, S. (2010). Predictors of bullying and victimization in childhood and adolescence: A meta‐analytic investigation. School Psychology Quarterly, 25, 65–83. 10.1037/a0020149

[ab21851-bib-0005] Ellis, B. J. , Del Giudice, M. , Dishion, T. J. , Figueredo, A. J. , Gray, P. , Griskevicius, V. , & Wilson, D. S. (2012). The evolutionary basis of risky adolescent behavior: Implications for science, policy, and practice. Developmental Psychology, 48, 598–623. 10.1037/a0026220 22122473

[ab21851-bib-0006] Ember, C. R. (1973). Feminine task assignment and the social behavior of boys. Ethos (Berkeley, Calif.), 1, 424–439. 10.1525/eth.1973.1.4.02a00050

[ab21851-bib-0007] Farmer, T. W. , Lines, M. M. , & Hamm, J. C. (2011). Revealing the invisible hand: The role of teachers in children's peer experiences. Journal of Applied Developmental Psychology, 32, 247–256. 10.1016/j.appdev.2011.04.006

[ab21851-bib-0008] Farris, R. , & Felmlee, D. (2011). Status struggles: Network centrality and gender segregation in same‐ and cross‐gender aggression. American Sociological Review, 76, 48–73. 10.1177/0003122410396196

[ab21851-bib-0009] Farris, R. , & Felmlee, D. (2014). Causalities of social combat: School networks of peer victimization and their consequences. American Sociological Review, 79, 228–257. 10.1177/0003122414524573

[ab21851-bib-0010] Gray, P. (2011). The special value of children's age‐mixed play. American Journal of Play, 3, 500–522.

[ab21851-bib-0011] Guerra, N. G. , Williams, K. R. , & Sadek, S. (2011). Understanding bullying and victimization during childhood and adolescence: A mixed‐method study. Child Development, 82, 295–310. 10.1111/j.1467-8624.2010.01556.x 21291443

[ab21851-bib-0012] Haltigan, J. D. , & Vaillancourt, T. (2018). The influence of static and dynamic intrapersonal factors on longitudinal patterns of peer victimization through mid‐adolescence: A latent transition analysis. Journal of Abnormal Child Psychology, 46, 11–26. 10.1007/s10802-017-0342-1 28861659

[ab21851-bib-0013] Huitsing, G. , Lodder, G. M. A. , Oldenburg, B. , Schacter, H. L. , Salmivalli, C. , Juvonen, J. , & Veenstra, R. (2019). The healthy context paradox: Victims’ adjustment during an anti‐bullying intervention. Journal of Family Studies, 10.1007/s10826-018-1194-1

[ab21851-bib-0014] Huitsing, G. , van Duijn, M. A. J. , Snijders, T. A. B. , Wang, P. , Sainio, M. , Salmivalli, C. , & Veenstra, R. (2012). Univariate and multivariate models of positive and negative networks: Liking, disliking, and bully‐victim relationships. Social Networks, 34, 645–657. 10.1016/j.socnet.2012.08.001

[ab21851-bib-0015] Huitsing, G. , & Veenstra, R. (2012). Bullying in classrooms: Participant roles from a social network perspective. Aggressive Behavior, 38, 494–509. 10.1002/ab.21438 22833443

[ab21851-bib-0016] Juvonen, J. (2018). The potential of schools to facilitate and constrain peer relationships In Bukowski W. M., Laursen B., & Rubin K. H. (Eds.), Handbook of peer interactions, relationships, and groups (pp. 491–509). New York: Guildford.

[ab21851-bib-0017] Juvonen, J. , & Graham, S. (2014). Bullying in schools: The power of bullies and the plight of victims. Annual Review of Psychology, 65, 159–185. 10.1146/annurev-psych-010213-115030 23937767

[ab21851-bib-0018] Kaufman, T. M. L. , Kretschmer, T. , Huitsing, G. , & Veenstra, R. (2018). Why does a universal anti‐bullying program not help all children? Explaining persistent victimization during an intervention. Prevention Science, 19, 822–832. 10.1007/s11121-018-0906-5 29707731

[ab21851-bib-0019] Kolbert, J. B. , & Crothers, L. M. (2003). Bullying and evolutionary psychology: The dominance hierarchy among students and implications for school personnel. Journal of School Violence, 2, 73–91. 10.1300/J202v02n03_05

[ab21851-bib-0020] Lee, K. , Dale, J. , Guy, A. , & Wolke, D. (2018). Bullying and negative appearance feedback among adolescents: Is it objective or misperceived weight that matters? Journal of Adolescence, 63, 118–128. 10.1016/j.adolescence.2017.12.008 29289824

[ab21851-bib-0021] Lillard, A. , & Else‐Quest, N. (2006). Evaluating Montessori education. Science, 313, 1893–1894. 10.1126/science.1132362 17008512

[ab21851-bib-0022] Lusher, D. , Koskinen, J. , & Robins, G. (2013). Exponential random graph models for social networks: Theory, methods, and applications. New York, NY: Cambridge University Press.

[ab21851-bib-0023] Maccoby, E. E. (1998). The two sexes: Growing apart, coming together. Cambridge, MA: Harvard University Press.

[ab21851-bib-0024] Mason, D. A. , & Burns, R. B. (1996). “Simply no worse and simply no better” may simply be wrong: A critique of Veenman's conclusion about multigrade classes. Review of Educational Research, 66, 307–322. 10.3102/00346543066003307

[ab21851-bib-0025] Miller, B. A. (1990). A review of the quantitative research on multigrade instruction. Research on Rural Education, 7, 1–8

[ab21851-bib-0026] Moller, A. C. , Forbes‐Jones, E. , & Hightower, A. D. (2008). Classroom age composition and developmental change in 70 urban preschool classrooms. Journal of Educational Psychology, 100, 741–753. 10.1037/a0013099

[ab21851-bib-0027] Mulryan‐Kyne, C. (2005). The grouping practices of teachers in small two‐teacher primary schools in the Republic of Ireland. Journal of Research on Rural Education, 20, 1–14

[ab21851-bib-0028] Mulryan‐Kyne, C. (2007). The preparation of teachers for multigrade teaching. Teaching and Teacher Education, 23, 501–514. 10.1016/j.tate.2006.12.003

[ab21851-bib-0029] Olweus, D. (1996). The revised Olweus bully/victim questionnaire. Bergen, Norway: Research Center for Health Promotion (HEMIL Center), University of Bergen.

[ab21851-bib-0030] Piaget, J. (1953). The origin of intelligence in the child. New Fetter Lane, New York: Routledge & Kegan Paul.

[ab21851-bib-0031] Pratt, D. (1986). On the merits of multiage classrooms. Research in Rural Education, 3, 111–115

[ab21851-bib-0032] Rambaran, J. A. , van Duijn, M. A. J. , Dijkstra, J. K. , & Veenstra, R. (2019). Stability and change in student classroom composition and its impact on peer victimization. Resubmitted for publication.

[ab21851-bib-0033] Rivara, F. , & Le Menestrel, S. (2016). Preventing Bullying Through Science, Policy, and Practice. Washington, DC: The National Academies Press. 27748087

[ab21851-bib-0034] Rivas‐Drake, D. , Saleem, M. , Schaefer, D. R. , Medina, M. , & Jagers, R. (2019). Intergroup contact attitudes across peer networks in school: Selection, influence and implications for cross‐group friendship. Child Development, 10.1111/cdev.13061 29785741

[ab21851-bib-0035] Rivers, I. , & Smith, P. (1994). Types of bullying behaviour and their correlates. Aggressive Behavior, 20, 359–368. 10.1002/1098-2337(1994)20:5<359::AID-AB2480200503>3.0.CO;2-J

[ab21851-bib-0036] Robins, G. (2015). Doing social network research: Network‐based research design for social scientists. London: Sage Publishing.

[ab21851-bib-0037] Rodkin, P. C. , Espelage, D. L. , & Hanish, L. D. (2015). A relational framework for understanding bullying: Developmental antecedents and outcomes. American Psychologist, 70, 311–321. 10.1037/a0038658 25961312

[ab21851-bib-0038] Rose, A. J. , & Rudolph, K. D. (2006). A review of sex differences in peer relationship processes: Potential trade‐offs for the emotional and behavioral development of girls and boys. Psychological Bulletin, 132, 98–131. 10.1037/0033-2909.132.1.98 16435959 PMC3160171

[ab21851-bib-0039] Salmivalli, C. (2010). Bullying and the peer group: A review. Aggression and Violent Behavior, 15, 112–120. 10.1016/j.avb.2009.08.007

[ab21851-bib-0040] Salmivalli, C. , Lagerspetz, K. , Björkqvist, K. , Österman, K. , & Kaukiainen, A. (1996). Bullying as a group process: Participant roles and their relations to social status within the group. Aggressive Behavior, 22, 1–15. 10.1002/(SICI)1098-2337(1996)22:1<1::AID-AB1>3.0.CO;2-T

[ab21851-bib-0041] Scheithauer, H. , Hayer, T. , Petermann, F. , & Jugert, G. (2006). Physical, verbal, and relational forms of bullying among German students: Age trends, gender differences, and correlates. Aggressive Behavior, 32, 261–275. 10.1002/ab.20128

[ab21851-bib-0042] Solberg, M. E. , & Olweus, D. (2003). Prevalence estimation of school bullying with the Olweus bully/victim questionnaire. Aggressive Behavior, 29, 239–268. 10.1002/ab.10047

[ab21851-bib-0043] Veenman, S. (1995). Cognitive and noncognitive effects of multigrade and multi‐age classes: A best‐evidence synthesis. Review of Educational Research, 65, 319–381. 10.3102/00346543065004319

[ab21851-bib-0044] Veenman, S. (1996). Effects of multgrade and multi‐age classes reconsidered. Review of Educational Research, 66, 323–340. 10.3102/00346543066003323

[ab21851-bib-0045] Veenstra, R. , Lindenberg, S. , Munniksma, A. , & Dijkstra, J. K. (2010). The complex relation between bullying, victimization, acceptance, and rejection: Giving special attention to status, affection, and sex differences. Child Development, 81, 480–486. 10.1111/j.1467-8624.2009.01411.x 20438454

[ab21851-bib-0046] Viechtbauer, W. (2010). Conducting meta‐analyses in R with the metafor package. Journal of Statistical Software, 36, 1–48. 10.18637/jss.v036.i03

[ab21851-bib-0047] Volk, A. A. , Camilleri, J. A. , Dane, A. V. , & Marini, Z. A. (2012). Is adolescent bullying an evolutionary adaptation? Aggressive Behavior, 38, 222–238. 10.1002/ab.21418 22331629

[ab21851-bib-0048] Wang, P. , Robins, G. , & Pattison, P. (2009). PNet: Program for the simulation and estimation of exponential random graph models. Melbourne School of Psychological Sciences, University of Melbourne http://www.melnet.org.au/pnet.

