# Supplementary material for: Peer victimization in single‐grade and multigrade classrooms
Source: Aggress Behav. 2019 Jun 26;45(5):561–70. doi: 10.1002/ab.21851 (PMC6772899; doi:10.1002/ab.21851)
Supplement: Supplementary file 1 — Supporting information [file AB-45-561-s001.doc]

Supporting Information to:

**‘Peer Victimization in Single-Grade and Multi-Grade Classrooms’**

Authored by:

Ashwin Rambaran, Marijtje van Duijn, Jan Kornelis Dijkstra, René Veenstra

List of Appendices, Tables and Figures:

**Appendix A.** Description of the selection criteria resulting in the final network sample.

**Figure A.** Schematic overview of the selection criteria resulting in the final network sample.

**Appendix B.** Differences between the full sample, reduced sample, and the final network sample.

**Table B1.** Differences between the full sample, reduced sample, and the final network sample.

**Table B2.** Network distribution differences between the reduced sample and the final network sample.

**Appendix C.** Victimization based on sex, grade, and age in the final network sample.

**Figure C1.** Distribution of classroom network density based on victimization by sex.

**Figure C2.** Distribution of classroom network density based on victimization by grade.

**Figure C3.** Summarized distribution of age-based victimization.

**Appendix D.** Results of the goodness of fit statistics.

**Table D1.** Goodness of fit statistics for each classroom victimization network model.

**Table D2.** Summary of goodness of fit statistics.

**Appendix E.** Results of the separate classroom ERGM analysis.

**Figure E.** Results of the separate classroom ERGM analysis – separate per effect and classroom.

**Appendix F.** ERGM meta-analysis results with outliers.

**Appendix G.** ERGM meta-analysis results with lower-bound victimization.

**Appendix H.** ERGM meta-analysis results – effects of sex-based age difference.

**Appendix A.** Description of the selection criteria resulting in the final network sample.

Figure A shows the selection criteria that were used in the present study. In the present study, KiVa data from the second wave were used (in October 2012). Schools were selected that had either only single-grade classrooms or only multi-grade classrooms to avoid potential differences due to a mixed-school setting. Seven schools in the control condition formed both single-grade classrooms and multi-grade classrooms and were excluded from the sample. Schools with multi-grade classrooms had either two or three grades in the same classroom. Because schools with only single-grade classrooms participated with grades 3-6, we excluded multi-grade schools with grades 1-2. As can be seen in Table B1, most multi-grade classrooms were grade 3-4 or grade 5-6 combinations. Therefore, we excluded multi-grade classrooms with a different grade combination. Because the remaining administrative multi-grade schools each had four grades (3-4 and 5-6), we excluded single-grade schools with more than 4 grades. Finally, we excluded classrooms with less than 15 students. Smaller classrooms are hard to compare to the more common larger classrooms and tend to carry less information which complicates the statistical social network analysis.

Criterion 3:

Eligible for social network analysis (>15 students in class, ref. cat. available) e

33 control schools (133 classes, 3,183 students)

7 mixed-school settings (39 classes, 1,008 students) a

13 administrative multi-grade (31 classes, 642 students)

8 regular single-grade (50 classes, 1,197 students)

3 with req. grades (12 classes, 295 students)

5 with multiple (>4) grades (38 classes, 902 students) b

6 with other grades (13 classes, 276 students) c

9 with req. grades (3-6) (18 classes, 366 students)

Criterion 1:

Either all single-grade or all multi-grade classes in school

Criterion 2:

All (4) grades fall in range of 3-6; multi-grade with two grades in class (3-4 & 5-6)

5 pedagogical multi-grade (12 classes, 308 students)

2 with req. grades (8 classes, 198 students) d

2 with other grades (4 classes, 110 students)

**Figure A.** Schematic overview of the selection criteria.

*Notes.* a=Schools with a mixed-setting had both single-grade classrooms (at least one) and multi-grade classrooms (at least one); b=Schools had multiple single-grades (> one grade 3, 4, 5 or 6); c=Schools had many different not so straightforward other combinations (e.g., 2-3, 4-5, 1-3, 4-6; see Table B1). To ensure comparability with single-grade classrooms (equal amount of four grades in schools: 3-6), we omitted these schools; d=One classroom (28 students) had a different combination (4-5) and was left out; e= e=six classrooms had incomplete sex combinations (1 single-grade; 2 administrative multi-grade; 1 pedagogical multi-grade) or grade (1 administrative multi-grade; 2 pedagogical multi-grades). Six other classrooms (all administrative multi-grades) contained less than 15 students.

3 with req. grades (11 classes, 274 students)

9 with req. grades 9 classes, 216 students)

2 with req. grades (6 classes, 156 students) d

Reduced

Reduced

Reduced

Network

Network

Network

**Appendix B.** Differences between the full sample, reduced sample, and the final network sample.

Table B1 provides an overview of differences between the full sample, reduced sample, and final network sample. Average density (proportion of nominations given) between the full and the final network sample was similar for both single-grade classrooms (.040 versus .038) and pedagogical multi-grade classrooms (.020 versus .021), but somewhat higher for administrative multi-grade classrooms (.031 versus .042). The reason for this is because school classrooms with grade 1 and/or grade 2 had low to zero density, lowering the average density in the full sample.

Table B2 shows an overview of the differences in network features between the reduced and the final network sample. As can be seen in Table B2, the distribution of ties varied between the networks within each classroom type (referring to regular single-grade, administrative multi-grade, and pedagogical multi-grade). It did not vary much between the three classroom types nor between the full sample and reduced sample. The same holds for basic structural networks patterns: The average numbers of sinks (referring to students who are bullies but not victims), sources (students who are victims but not bullies), and isolates (referring to students who are neither victims nor bullies) were similar between the two samples. More complex structural network patterns – geodesic distances (indirect ties), reciprocity (bully-victim ties), and transitivity (cohesion) – were hardly present in the data. This is common for negative networks in general and peer victimization networks in specific (Huitsing et al., 2012; Huitsing & Veenstra, 2012). This indicates that victimization ties were mostly directed and unilateral. In sum, overall the differences between the two samples were not large.

| **Table B1.** Overview of differences between the full sample, reduced sample, and the final network samples. | | | | | | | | | | | | | | | | |
| --- | --- | --- | --- | --- | --- | --- | --- | --- | --- | --- | --- | --- | --- | --- | --- | --- |
|  | | **Full sample (33 schools, 133 classrooms)** | | | | | **Reduced sample (14 schools, 38 classrooms)** | | | | | **Network sample (12 schools, 26 classrooms)** | | | | |
| **Sample description** | | No. of students | No. of classes | M class size | Av. % boys | Av. density | No. of students | No. of classes | M class size | Av. % boys | Av. density | No. of students | No. of classes | M class size | Av. % boys | Av. density |
| Regular single-grade | | 1,807 | 74 | 24 | 50 | .040 | 295 | 12 | 25 | 49 | .036 | 274 | 11 | 25 | 48 | .038 |
|  | Grade 3 | 450 | 18 | 25 | 49 | .047 | 74 | 3 | 25 | 45 | .025 | 74 | 3 | 25 | 45 | .025 |
|  | Grade 4 | 436 | 18 | 24 | 46 | .046 | 84 | 3 | 29 | 44 | .038 | 84 | 3 | 28 | 44 | .038 |
|  | Grade 5 | 416 | 17 | 24 | 52 | .041 | 66 | 3 | 23 | 55 | .062 | 66 | 3 | 22 | 55 | .062 |
|  | Grade 6 | 505 | 21 | 24 | 53 | .030 | 71 | 3 | 24 | 55 | .019 | 50 | 2 | 25 | 52 | .022 |
| Administrative multi-grade | | 1,040 | 46 | 23 | 50 | .031 | 366 | 18 | 20 | 51 | .049 | 216 | 9 | 24 | 52 | .042 |
|  | Grade 1-3 | 22 | 1 | 22 | 55 | .000 | - | - | - | - | - | - | - | - | - | - |
|  | Grade 2-3 a | 89 | 4 | 22 | 54 | .015 | - | - | - | - | - | - | - | - | - | - |
|  | Grade 2-4 a | 27 | 1 | 27 | 22 | .028 | - | - | - | - | - | - | - | - | - | - |
|  | Grade 3-4 | 263 | 13 | 20 | 48 | .054 | 160 | 9 | 18 | 48 | .066 | 88 | 4 | 22 | 49 | .052 |
|  | Grade 4-5 | 133 | 5 | 27 | 50 | .029 | - | - | - | - | - | - | - | - | - | - |
|  | Grade 5-6 | 407 | 17 | 24 | 53 | .026 | 206 | 9 | 23 | 54 | .033 | 128 | 5 | 26 | 55 | .033 |
|  | Grade 1-2-3 a | 16 | 1 | 16 | 56 | .000 | - | - | - | - | - | - | - | - | - | - |
|  | Grade 2-3-4 a | 17 | 1 | 17 | 24 | .000 | - | - | - | - | - | - | - | - | - | - |
|  | Grade 4-5-6 | 66 | 3 | 22 | 48 | .017 | - | - | - | - | - | - | - | - | - | - |
| Pedagogical multi-grade | | 336 | 13 | 26 | 46 | .020 | 198 | 8 | 25 | 47 | .018 | 156 | 6 | 26 | 51 | .021 |
|  | Grade 3-4 | 94 | 4 | 24 | 50 | .025 | 94 | 4 | 24 | 54 | .025 | 78 | 3 | 26 | 51 | .030 |
|  | Grade 4-5 | 28 | 1 | 28 | 39 | .012 | - | - | - | - | - | - | - | - | - | - |
|  | Grade 5-6 | 104 | 4 | 26 | 44 | .011 | 104 | 4 | 26 | 44 | .011 | 78 | 3 | 26 | 50 | .012 |
|  | Grade 1-2-3 a | 23 | 1 | 23 | 39 | .006 | - | - | - | - | - | - | - | - | - | - |
|  | Grade 4-5-6 | 87 | 3 | 29 | 46 | .032 | - | - | - | - | - | - | - | - | - | - |
| *Notes.* a=No data available for Grade 1 and Grade 2 because they did not participate in the study. Accordingly, the low average densities for these multi-grade classrooms may be due to absent network data, and therefore these classrooms cannot be used for comparison. Figure A in Appendix A explains the difference between the reduced and network sample. | | | | | | | | | | | | | | | | |

| **Table B2.** Overview of network distribution differences between the reduced sample and the final network samples. | | | | | | | |
| --- | --- | --- | --- | --- | --- | --- | --- |
|  | | **Reduced sample** | | | **Network sample** | | |
|  | | Regular  single-grade (12 classes) | Administrative  multi-grade (18 classes) | Pedagogical  multi-grade (8 classes) | Regular  single-grade (11 classes) | Administrative  multi-grade (9 classes) | Pedagogical  multi-grade (6 classes) |
| ***Skewness*** (min-max) | |  |  |  |  |  |  |
|  | Av. in/out-degree | 0.8 (0.2-2.0) | 0.8 (0.0-1.7) | 0.4 (0.2-1.1) | 0.9 (0.2-2.0) | 0.9 (0.4-1.7) | 0.5 (0.2-1.1) |
|  | Av. st.dev. in-degree | 1.2 (0.5-2.3) | 1.1 (0.4-2.4) | 0.8 (0.5-1.4) | 1.2 (0.5-2.3) | 1.3 (0.8-2.3) | 0.9 (0.5-1.4) |
|  | Av. st.dev. out-degree | 1.6 (0.6-3.0) | 2.0 (0.9-4.0) | 0.9 (0.4-1.9) | 1.6 (0.6-3.0) | 2.5 (1.7-4.0) | 1.0 (0.5-1.9) |
|  | Av. skew of in-degree | 1.6 (-0.1-2.4) | 1.4 (0.0-2.6) | 2.5 (1.6-3.3) | 1.5 (-0.1-2.4) | 1.6 (0.7-2.6) | 2.4 (1.7-3.3) |
|  | Av. skew of out-degree | 2.2 (0.8-4.4) | 2.1 (1.1-4.7) | 2.4 (1.6-3.3) | 2.1 (0.8-4.4) | 2.2 (1.1-3.0) | 2.4 (1.7-3.3) |
| ***Structural configurations*** (min-max) | |  |  |  |  |  |  |
|  | Av. no. of sinks | 7 (4-12) | 6 (3-14) | 5 (2-9) | 7 (4-12) | 7 (3-11) | 6 (3-9) |
|  | Av. no. of sources | 4 (1-9) | 4 (1-8) | 6 (3-12) | 5 (1-9) | 5 (2-8) | 7 (4-12) |
|  | Av. no. of isolates | 11 (3-16) | 8 (0-20) | 12 (3-22) | 10 (3-16) | 9 (3-20) | 11 (3-22) |
| ***Geodesic distances*** (min-max) | |  |  |  |  |  |  |
|  | Av. 0 intermediaries (direct ties) | 20 (5-39) | 16 (2-38) | 10 (3-24) | 21 (5-39) | 21 (11-38) | 12 (5-24) |
|  | Av. 1 intermediary (indirect ties) | 9 (0-52) | 8 (0-38) | 4 (0-16) | 10 (0-52) | 15 (0-38) | 6 (0-16) |
|  | Av. 2 intermediaries | 2 (0-20) | 1 (0-13) | 0 (0-2) | 3 (0-20) | 3 (0-13) | 1 (0-2) |
|  | Av. 3 intermediaries | 1 (0-8) | 0 (0-1) | 0 (0-0) | 1 (0-8) | 0 (0-1) | 0 (0-0) |
| ***Reciprocity*** (min-max) | |  |  |  |  |  |  |
|  | Av. no. of reciprocity (mutual ties) | 1 (0-5) | 1 (0-3) | 0 (0-1) | 1 (0-5) | 1 (0-3) | 0 (0-1) |
| ***Transitivity*** (min-max) | |  |  |  |  |  |  |
|  | Av. transitivity (%) | 3.2 (0.0-14.1) | 2.3 (0.0-7.41) | 2.0 (0.0-6.3) | 3.5 (0.0-14.1) | 3.0 (0.0-7.41) | 2.7 (0.0-6.3) |
| *Notes.* Sinks (actors with zero out-degree). Sources (actors with zero in-degree). Isolates (actors with zero out-degree and zero in-degree); percentages may not match due to rounding differences. Geodesic distance = shortest path between two nodes; The density of transitive triples is the number of triples (of any form; see Wasserman & Faust, 1994) which are transitive divided by the number of paths of length 2, i.e. the number of triples which have the potential to be transitive. Transitivity was calculated in Ucinet 6 version 6.459 (Borgatti, Everett, & Freeman, 2002). | | | | | | | |

**Appendix C.** Results of goodness of fit statistics.

The results of the goodness of fit (GoF) statistics for each classroom victimization network model is presented in Table C1 and summarized in Table C2. The majority of the classroom victimization networks were adequately modeled by the effects reported in Table 2 in the paper. This means no additional network or individual effects in the model were needed to capture the relational patterns in these victimization networks, most likely because victimization networks are sparse networks. Accordingly, a small set of network effects suffices (referring to density, sinks, isolates, in-ties spread, multiple two-paths, and shared in-ties; see Huitsing et al., 2012; see also Huitsing & Veenstra, 2012).

In one classroom (3), the GoF statistics indicated that reciprocity was not captured adequately. In addition, in almost half of the classrooms (1, 7, 8, 9, 12, 13, 15, 18, 20, 23, 24, 26) the GoF statistics indicated that reciprocity with regard to age (in various forms, referring to sum, difference, product) was not modeled adequately. However, these configurations were only present in four of these classrooms (7, 8, 12, 23). Such configurations were not related to one particular effect, and because we already included non-reciprocal age-related effects to test our hypothesis, we decided not to include these extra effects.

| **Table C1.** Overview of goodness of fit (*t*) statistics for each classroom victimization network model seperately. | | | | | | | | | | | | | | | | | | | | | | | | | | |
| --- | --- | --- | --- | --- | --- | --- | --- | --- | --- | --- | --- | --- | --- | --- | --- | --- | --- | --- | --- | --- | --- | --- | --- | --- | --- | --- |
|  | Regular single-grade | | | | | | | | | | | Administrative multi-grade | | | | | | | | | Pedagogical multi-grade | | | | | |
|  | Grade 3-4 | | | | | | Grade 5-6 | | | | | Grade 3-4 | | | | Grade 5-6 | | | | | Grade 3-4 | | | Grade 5-6 | | |
| Statistic | 1 | 2 | 3 | 4 | 5 | 6 | 7 | 8 | 9 | 10 | 11 | 12 | 13 | 14 | 15 | 16 | 17 | 18 | 19 | 20 | 21 | 22 | 23 | 24 | 25 | 26 |
| *Network effects* |  |  |  |  |  |  |  |  |  |  |  |  |  |  |  |  |  |  |  |  |  |  |  |  |  |  |
| Arc | 0.00 | 0.06 | -0.02 | -0.08 | 0.01 | 0.12 | 0.03 | 0.00 | -0.20 | 0.02 | -0.02 | -0.05 | -0.03 | -0.05 | -0.02 | 0.00 | 0.05 | 0.05 | -0.18 | 0.03 | -0.08 | -0.01 | -0.04 | 0.01 | 0.04 | 0.00 |
| Reciprocity | -0.08 | 1.38 | 2.03 | -0.43 | 0.72 | 2.01 | 0.54 | 1.00 | -0.63 | -0.12 | -0.24 | 0.14 | -0.09 | 1.22 | -0.75 | -0.40 | 0.06 | -0.27 | 0.06 | -0.68 | -0.38 | -0.08 | 0.59 | -0.25 | -0.25 | -0.03 |
| 2-In-Star | 0.36 | 0.11 | 0.18 | -0.15 | 0.22 | 0.08 | 0.06 | -0.11 | -0.16 | 0.67 | -0.16 | -0.12 | -0.12 | 0.05 | -0.05 | -0.19 | 0.11 | 0.03 | -0.22 | -0.14 | -0.10 | -0.18 | 0.16 | 0.36 | -0.01 | -0.02 |
| 2-Out-Star | -0.29 | 0.07 | -0.09 | 0.16 | 0.00 | 0.10 | 0.02 | 0.02 | -0.14 | -0.25 | -0.04 | -0.10 | -0.05 | 0.01 | 0.03 | 0.40 | 0.06 | -0.02 | -0.20 | 0.05 | -0.09 | -0.35 | 0.12 | -0.05 | -0.02 | 0.21 |
| 3-In-Star | -0.17 | 0.15 | 0.37 | -0.27 | 0.61 | -0.02 | 0.21 | -0.39 | -0.36 | -0.13 | -0.37 | -0.22 | -0.26 | 0.36 | -0.15 | -0.51 | 0.24 | 0.07 | -0.35 | -0.48 | -0.12 | -0.43 | 0.56 | -0.28 | -0.09 | -0.19 |
| 3-Out-Star | -0.45 | -0.03 | -0.23 | 0.50 | -0.15 | 0.09 | -0.08 | -0.06 | -0.24 | -0.42 | -0.13 | -0.19 | -0.16 | -0.20 | -0.04 | 0.53 | -0.08 | -0.11 | -0.33 | -0.03 | -0.16 | -0.47 | 0.09 | -0.29 | -0.13 | 0.17 |
| Mixed-2-Star | -0.49 | -0.01 | -0.04 | -0.13 | -0.01 | 0.10 | 0.02 | -0.03 | -0.18 | -0.54 | -0.05 | -0.09 | -0.30 | -0.05 | -0.03 | 0.00 | 0.05 | -0.02 | -0.22 | -0.01 | -0.10 | -0.56 | -0.11 | -0.76 | 0.00 | -0.30 |
| 030T | -0.10 | 0.63 | 0.18 | 1.04 | -0.49 | 0.70 | 0.35 | -0.01 | 0.73 | -0.23 | -0.50 | -0.18 | -0.24 | 0.51 | -0.06 | -0.77 | 0.15 | -0.21 | -0.49 | 0.19 | -0.05 | -0.31 | 0.45 | -0.24 | 0.14 | -0.19 |
| 030C | -0.05 | -0.23 | -0.21 | -0.13 | -0.03 | 0.75 | -0.39 | 0.40 | -0.10 | -0.03 | -0.06 | -0.28 | -0.05 | -0.48 | -0.47 | -0.10 | -0.35 | -0.05 | -0.55 | -0.33 | -0.30 | -1.00 | -0.42 | -0.08 | -0.18 | -1.00 |
| Sink | -0.03 | 0.03 | -0.05 | -0.13 | 0.01 | 0.01 | 0.02 | -0.06 | -0.19 | -0.02 | 0.01 | 0.00 | -0.01 | 0.03 | 0.01 | -0.05 | 0.03 | 0.05 | -0.05 | 0.04 | -0.02 | -0.01 | -0.01 | 0.01 | 0.07 | -0.03 |
| Source | 0.28 | 0.10 | -0.23 | 0.13 | -0.57 | -0.06 | -0.21 | 0.65 | -0.02 | 0.43 | 0.11 | 0.24 | 0.24 | -0.09 | -0.15 | 0.37 | -0.15 | 0.06 | -0.38 | 0.59 | -0.22 | 0.31 | -0.20 | 0.61 | 0.00 | 0.01 |
| Isolates | 0.02 | -0.04 | 0.02 | 0.12 | 0.01 | -0.09 | -0.05 | 0.04 | 0.22 | 0.02 | 0.01 | 0.04 | 0.02 | 0.03 | 0.04 | 0.02 | -0.05 | -0.04 | 0.18 | -0.02 | 0.05 | 0.00 | 0.03 | 0.01 | -0.06 | 0.07 |
| K-In-Star | 0.44 | 0.08 | 0.02 | -0.06 | -0.01 | 0.12 | 0.01 | 0.00 | -0.15 | 0.74 | -0.03 | -0.05 | -0.03 | -0.05 | -0.02 | 0.00 | 0.04 | 0.01 | -0.17 | 0.02 | -0.10 | -0.01 | -0.05 | 0.55 | 0.03 | 0.08 |
| K-Out-Star | -0.18 | 0.09 | -0.06 | -0.01 | 0.06 | 0.10 | 0.04 | 0.01 | -0.12 | -0.13 | -0.01 | -0.05 | -0.01 | 0.15 | 0.03 | 0.25 | 0.11 | 0.04 | -0.13 | 0.07 | -0.02 | -0.22 | 0.05 | 0.00 | 0.04 | 0.19 |
| K-L-Star | -0.53 | -0.01 | 0.00 | -0.26 | 0.14 | 0.28 | 0.06 | -0.20 | -0.13 | -0.60 | -0.10 | -0.07 | -0.46 | -0.09 | -0.12 | -0.18 | 0.16 | 0.08 | -0.36 | -0.03 | 0.01 | -0.63 | -0.16 | -0.83 | 0.20 | -0.38 |
| K-1-Star | -0.49 | 0.27 | -0.19 | -0.59 | 0.04 | 0.14 | 0.23 | 0.02 | -0.26 | -0.55 | -0.04 | -0.06 | -0.38 | -0.16 | 0.10 | -0.39 | 0.11 | -0.06 | -0.17 | -0.17 | 0.05 | -0.59 | 0.13 | -0.79 | 0.17 | -0.35 |
| 1-L-Star | -0.53 | -0.25 | 0.16 | 0.36 | 0.08 | 0.26 | -0.14 | -0.26 | 0.01 | -0.58 | -0.04 | -0.05 | -0.36 | 0.01 | -0.23 | 0.38 | 0.34 | 0.10 | -0.26 | 0.17 | 0.01 | -0.59 | -0.42 | -0.79 | 0.03 | -0.33 |
| TK-Triangle | -0.10 | 0.72 | 0.20 | 1.19 | -0.49 | 0.73 | 0.28 | 0.08 | 0.75 | -0.23 | -0.50 | -0.15 | -0.26 | 0.48 | -0.11 | -0.79 | 0.23 | -0.19 | -0.49 | 0.24 | -0.02 | -0.31 | 0.65 | -0.24 | 0.19 | -0.19 |
| CK-Triangle | -0.05 | -0.23 | -0.21 | -0.13 | -0.03 | 0.90 | -0.40 | 0.40 | -0.10 | -0.03 | -0.06 | -0.29 | -0.05 | -0.49 | -0.49 | -0.10 | -0.38 | -0.05 | -0.57 | -0.33 | -0.31 | -1.00 | -0.43 | -0.08 | -0.19 | -1.00 |
| DK-Triangle | -0.10 | 0.78 | 0.23 | 0.19 | -0.49 | 0.66 | 0.38 | 0.12 | 0.73 | -0.23 | -0.47 | -0.14 | -0.28 | 0.47 | -0.02 | -0.79 | 0.14 | -0.17 | -0.49 | 0.24 | -0.07 | -0.31 | 0.40 | -0.24 | 0.28 | -0.20 |
| UK-Triangle | -0.10 | 0.41 | 0.20 | 1.41 | -0.49 | 0.76 | 0.37 | -0.03 | 0.65 | -0.23 | -0.49 | -0.15 | -0.28 | 0.55 | -0.17 | -0.79 | 0.13 | -0.19 | -0.51 | 0.06 | 0.05 | -0.31 | 0.22 | -0.24 | 0.21 | -0.19 |
| TK-2-Paths | -0.49 | 0.03 | -0.03 | -0.09 | 0.00 | 0.11 | 0.01 | 0.01 | -0.14 | -0.54 | -0.02 | -0.05 | -0.31 | -0.05 | 0.01 | 0.03 | 0.08 | 0.02 | -0.19 | 0.04 | -0.09 | -0.57 | -0.01 | -0.76 | 0.01 | -0.30 |
| DK-2-Paths | -0.29 | 0.07 | -0.04 | -0.09 | 0.04 | 0.11 | 0.01 | 0.00 | -0.13 | -0.24 | 0.00 | -0.05 | -0.02 | -0.07 | -0.01 | -0.01 | 0.07 | 0.07 | -0.19 | 0.06 | -0.08 | -0.34 | -0.04 | -0.04 | 0.04 | 0.25 |
| UK-2-Paths | 0.37 | 0.11 | 0.22 | -0.35 | 0.29 | 0.10 | -0.04 | -0.12 | -0.16 | 0.68 | -0.15 | -0.08 | -0.08 | -0.17 | -0.11 | -0.55 | 0.18 | 0.15 | -0.22 | -0.14 | -0.08 | -0.16 | -0.01 | 0.38 | 0.05 | -0.01 |
| *Age effects* |  |  |  |  |  |  |  |  |  |  |  |  |  |  |  |  |  |  |  |  |  |  |  |  |  |  |
| Sender | -0.05 | 0.03 | -0.03 | -0.09 | -0.02 | 0.06 | -0.10 | 0.01 | 0.19 | 0.03 | 0.09 | 0.02 | 0.03 | 0.01 | 0.00 | -0.01 | -0.03 | -0.04 | 0.08 | -0.01 | 0.14 | -0.01 | 0.02 | -0.07 | -0.03 | -0.01 |
| Receiver | 0.00 | -0.07 | 0.04 | 0.02 | -0.02 | 0.14 | 0.05 | -0.04 | 0.07 | -0.03 | 0.04 | 0.06 | 0.05 | 0.08 | 0.04 | -0.01 | -0.06 | 0.05 | 0.16 | -0.05 | 0.05 | -0.01 | 0.01 | 0.10 | 0.07 | 0.01 |
| Single-Sum | -0.03 | -0.04 | 0.00 | -0.06 | -0.04 | 0.12 | -0.08 | 0.00 | 0.22 | 0.01 | 0.12 | 0.04 | 0.04 | 0.04 | 0.02 | -0.02 | -0.05 | -0.01 | 0.13 | -0.02 | 0.11 | -0.02 | 0.02 | 0.04 | 0.02 | 0.00 |
| Single-Difference | -0.01 | -0.08 | 0.82 | 0.19 | -0.10 | 0.31 | -0.42 | 0.12 | -0.40 | -0.32 | 0.39 | -0.14 | -0.18 | -0.64 | -0.51 | -0.16 | -0.30 | -0.15 | -0.46 | 0.24 | -0.41 | -0.47 | -0.60 | -0.85 | 0.22 | -0.43 |
| Single-Product | -0.72 | -0.04 | -2.13 | -0.25 | -0.57 | 0.69 | 0.40 | -0.67 | -0.40 | 0.98 | -0.52 | -0.19 | 0.02 | 0.10 | 0.21 | -0.13 | 0.17 | 0.66 | 0.12 | -0.04 | 0.09 | 0.36 | 0.15 | 0.91 | -0.24 | -0.01 |
| Mutual-Sum | 1.45 | -0.39 | -0.08 | 0.10 | -1.46 | -1.21 | -1.31 | 1.35 | 1.64 | -2.33 | -0.81 | -0.96 | -1.42 | 1.74 | 0.82 | 0.65 | 1.03 | -0.90 | -1.63 | 0.87 | 2.62 | -1.16 | 1.82 | -0.01 | -0.73 | -2.27 |
| Mutual-Difference | 1.51 | -1.75 | -1.98 | -1.67 | -2.54 | -1.55 | -0.18 | -0.17 | -1.49 | -1.67 | -1.47 | 2.28 | -1.54 | -0.32 | -0.16 | -1.09 | -0.46 | 0.32 | 0.68 | 1.32 | 2.43 | -2.76 | -1.66 | -0.59 | 1.29 | -2.68 |
| Mutual-Product | -1.41 | 1.75 | 3.41 | 1.60 | 1.87 | 1.06 | -1.15 | 1.06 | 0.85 | 0.20 | -0.58 | -1.41 | 1.78 | -1.75 | -1.19 | 0.48 | -0.91 | 0.45 | -0.56 | -0.14 | -2.45 | 2.45 | 0.07 | 0.26 | 0.74 | 3.09 |
| Relative Difference | -0.03 | 0.06 | -0.05 | -0.10 | 0.00 | -0.12 | -0.11 | 0.03 | 0.16 | 0.04 | 0.01 | -0.07 | 0.00 | -0.04 | -0.03 | -0.01 | 0.06 | -0.08 | -0.09 | 0.04 | 0.12 | 0.00 | 0.01 | -0.11 | -0.07 | -0.02 |
| *Sex effects* |  |  |  |  |  |  |  |  |  |  |  |  |  |  |  |  |  |  |  |  |  |  |  |  |  |  |
| Boy-Boy | 0.88 | 1.27 | 1.93 | 4.00 | 0.02 | 1.13 | 0.50 | -0.03 | 6.39 | 1.32 | 0.25 | 1.13 | 0.03 | 0.00 | 0.43 | 0.99 | 0.04 | 1.22 | -0.20 | -0.01 | -0.08 | -0.01 | -0.05 | 1.29 | 0.02 | 1.71 |
| Girl-Girl | 0.03 | -0.05 | 0.02 | -1.26 | 0.05 | 0.10 | -0.85 | 0.00 | -1.51 | -0.01 | -0.92 | -0.99 | -0.04 | -0.06 | -1.26 | 0.04 | 0.03 | 0.02 | -0.08 | -0.01 | -0.02 | -0.04 | -0.03 | -0.67 | 0.04 | 0.09 |
| Girl-Boy | 0.00 | 0.08 | -0.02 | -0.06 | -0.02 | 0.12 | 0.04 | 0.00 | -0.20 | -1.09 | -0.01 | -0.06 | -0.01 | -0.04 | 0.00 | 0.05 | 0.04 | 0.03 | -0.12 | 0.05 | -0.06 | 0.02 | 0.00 | 0.03 | 0.04 | -0.04 |
| Boy-Girl | -0.79 | -1.42 | -1.63 | -0.52 | -0.05 | -1.39 | 0.00 | 0.02 | -0.84 | 0.01 | 0.00 | -0.08 | -0.03 | -0.01 | 0.01 | -0.98 | 0.05 | -1.36 | -0.18 | 0.06 | -0.07 | -0.01 | -0.02 | -0.91 | 0.02 | -1.12 |
| *Grade effects* |  |  |  |  |  |  |  |  |  |  |  |  |  |  |  |  |  |  |  |  |  |  |  |  |  |  |
| Low-Low | -- | -- | -- | -- | -- | -- | -- | -- | -- | -- | -- | -0.05 | 0.73 | -0.04 | 0.03 | 1.39 | 0.02 | 0.01 | 0.89 | 0.02 | -0.09 | 1.25 | 1.63 | 0.04 | 0.03 | 1.60 |
| High-High | -- | -- | -- | -- | -- | -- | -- | -- | -- | -- | -- | -0.07 | -0.06 | 0.04 | 0.00 | -0.02 | 0.08 | 0.03 | -0.82 | 0.02 | 0.00 | -0.64 | -0.01 | -0.12 | 0.02 | 0.04 |
| Low-High | -- | -- | -- | -- | -- | -- | -- | -- | -- | -- | -- | -0.04 | -0.54 | -0.06 | -0.08 | 0.03 | 0.06 | 0.00 | -0.17 | 0.00 | -0.10 | -0.03 | -1.37 | -0.04 | 0.05 | 0.03 |
| High-Low | -- | -- | -- | -- | -- | -- | -- | -- | -- | -- | -- | 0.01 | 0.02 | 0.00 | 0.02 | -0.81 | 0.02 | 0.05 | -0.18 | 0.02 | -0.02 | -0.76 | -0.02 | 0.05 | 0.03 | -1.01 |
| *Notes.* The table shows the t-statistics for assessment of goodness of fit for each effect included and not included in the model. The t-statistic is calculated with (observation - sample mean) / standard deviation. | | | | | | | | | | | | | | | | | | | | | | | | | | |

| **Table C2.** Summary of goodness of fit results. | | | |
| --- | --- | --- | --- |
| 11 Regular single-grade classrooms | 9 Administrative multi-grade classrooms | 6 Pedagogical multi-grade classrooms | Summary/conclusion |
| 9 classrooms showed good fit statistics.^a^ | 8 classrooms showed good fit statistics.^a^ | 5 classrooms showed good fit statistics.^a^ | The majority of victimization networks were adequately modeled by included effects. |
| 2 classrooms (3 and 6) showed poor fit on reciprocity effect. |  |  | Reciprocity (*i*↔*j*) was not adequately modeled in two classrooms. Because there are a few of such ties in these classrooms, it was not modeled explicitly. |
| 3 classrooms (3, 5, and 10) showed poor fit on the mutual age-sum/product effect. | 1 classroom (12) showed poor fit on the mutual age sum/difference effect. | 3 classrooms (21, 22, and 26) showed poor fit on the mutual age sum/product effect. | Reciprocity with regard to age effects was not adequately modeled in several classrooms. Because we already included non-reciprocal age effects, and it was not related to one particular age effect, no additional age effects were included. |
| *Notes.* ^a^Good/acceptable fit is indicated by t-statistics lower than 2 in absolute terms for effects not included in the model. | | | |

**Appendix D.** Victimization based on sex, grade, and age in the final network sample.

**Sex.** Figure D1 shows an overview of the distribution of the classroom network density based on sex, sorted by classroom network density (lowest to highest) and classroom type (referring to regular single-grade, administrative multi-grade, and pedagogical multi-grade). The figure above shows the densities per classroom for the younger cohort group (grade 3-4); the figure below shows the densities for the older cohort group (grade 5-6). Victimization among boys (‘boy victimize boy’) occurred in every classroom, whereas victimization among girls occurred in most but not all school classrooms. In most classrooms, victimization was higher among boys than among girls (with a few exceptions). Also, in most classrooms, boys victimized girls more than vice versa (with a few exceptions).

**Grade.** Figure D2 shows an overview of the distribution of the classroom network density based on grade, again sorted by classroom network density and classroom type. In most classrooms victimization occurred among same-grade students (with some exceptions). In addition, victimization was somewhat higher among the younger cohort group (Grade 3-4) than among the older cohort group (Grade 5-6). In half of the classrooms (7 times) higher-grade students victimized lower-grade students more than vice versa; the opposite was true in the other half of the classrooms (5 times); in three classrooms higher-grade students and lower-grade classrooms victimized each other to an equal extent.

**Age.** Figure D3 shows the summarized distribution of victimization based on age, separated by cohort and classroom type. The figure shows the summarized differences in age between victims’ bullies and victims’ non-bullies. The dashed line represents the mean age in a classroom. The part above and below the dashed line represent respectively children who are being victimized by older and younger children in classroom (relative to those by whom they are not victimized). The difference centers around zero which indicates that children who bully others are not older than children who do not bully others.

**Figure C1.** Distribution of classroom network density based on victimization by boys and girls, sorted by classroom network density (lowest to highest) and classroom type (regular single-grade: 1-11; administrative multi-grade: 12-20; pedagogical multi-grade: 21-26). Figure above shows the densities per classroom for the younger cohort group (grade 3-4 = 1-6, 12-15, 21-23), Figure below shows the densities for the older cohort group (grade 5-6 = 7-11, 16-20, 24-26).

*Notes.* The density for each group was calculated as the number of observed ties in each group divided by the total number of possible ties in each group. Density was calculated in Ucinet 6 version 6.459 (Borgatti, Everett, & Freeman, 2002).

**Figure C2.** Distribution of classroom network density based on victimization by higher-grade students and lower-grade students, sorted by classroom network density (lowest to highest) and classroom type (administrative multi-grade: 12-20; pedagogical multi-grade: 21-26). The top figure shows the densities per classroom for the younger cohort group (grade 3-4 = 12-15, 21-23), The bottom figure shows the densities for the older cohort group (grade 5-6 = 16-20, 24-26).

*Notes.* The density for each group was calculated as the number of observed ties in each group divided by the total number of possible ties in each group. Density was calculated in Ucinet 6 version 6.459 (Borgatti, Everett, & Freeman, 2002). The average per classroom type is presented in the final colum for each classroom type.


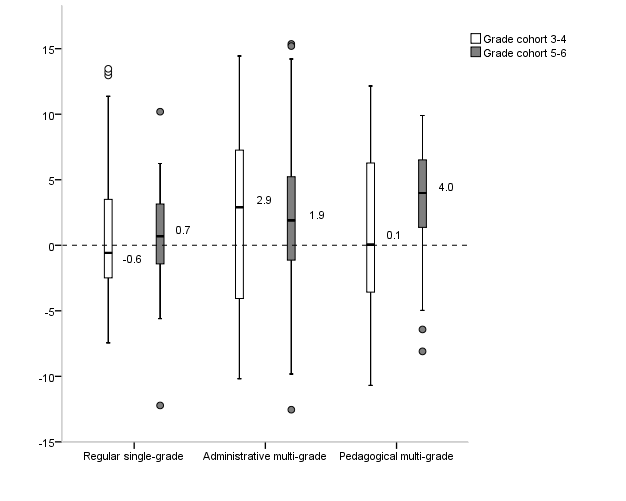
**Figure C3.** Summarized distribution of age-based victimization separated by grade cohort (grade 3-4, grade 5-6) and classroom type (regular single-grade, administrative multi-grade, pedagogical multi-grade).

*Notes.* The figure shows the summarized differences in age (*a*) between victims’ bullies (*j*) and victims’ non-bullies (*jˊ*), calculated as: $\sum_{j} a_{\bar{\mathrm{bj}}}-\sum_{jˊ} a_{\mathrm{bj}}$ , and separated by grade cohort. The dashed line in the middle represents the mean age in classroom. The part of the bar *above* the dashed line represents the children who are being victimized by older children in classroom, whereas the part *below* the dashed line represents the children who are being victimized by younger children in classroom.

**Appendix E.** Results of the separate classroom ERGM analysis – separate per effect and classroom.

Appendix E shows the results per classroom with forest plots (cf. Viechtbauer, 2010). We use them to identify potential outliers. Visual inspection reveals two potentially influential cases. Single-grade classroom 9 is clearly an outlier, with a positive density (see Figure A). This is because every boy in class was nominated as bully and boy-boy is the reference category. In addition, administrative multi-grade classroom 17 is also suspicious with a very negative high-low effect (see Figure L). Additional analysis (using build-in diagnostics in metafor; c.f. Viechtbauer & Cheung, 2010) showed that these two classrooms were indeed outliers.

| **Appendix F*.*** ERGM meta-analysis results for victimization networks in single-grade and multi-grade classrooms results with outliers (classroom 9 and 17). | | | | | | | | |
| --- | --- | --- | --- | --- | --- | --- | --- | --- |
|  |  |  | ***Intercept (administrative multi-grade)*** | | ***Intercept +***  ***regular single-grade*** | | ***Intercept +***  ***pedagogical***  ***multi-grade*** | |
| Parameter | *Illustratation* | *n* | *Est.* | *(S.E.)* | *Est.* | *(S.E.)* | *Est.* | *(S.E.)* |
| ***Network effects*** |  |  |  |  |  |  |  |  |
| Density (Arc) |  | 26 | -3.21*** | (0.80)^a^ | 1.06 | (1.08)^a^ | -1.06 | (1.27)^a^ |
| Sinks (sink) |  | 26 | 1.04* | (0.18) | 0.18 | (0.62) | 0.32 | (0.74) |
| Isolates (isolates) |  | 26 | 1.28+ | (0.69)^a^ | 1.30 | (0.95)^a^ | 0.07 | (1.11)^a^ |
| In-ties spread (AinS) |  | 23 | 0.73 | (0.56)^a^ | -1.00 | (0.79)^a^ | -0.14 | (0.96)^a^ |
| Multiple two-paths (A2P-T) |  | 20 | -0.01 | (0.15) | -0.14 | (0.22) | 0.15 | (0.30) |
| Shared in-ties (A2P-D) |  | 21 | 0.15 | (0.14) | -0.01 | (0.18) | -0.04 | (0.28) |
| ***Sex effects*** |  |  |  |  |  |  |  |  |
| Boy-boy (ref.cat.) |  | 26 | -- | -- | -- | -- | -- | -- |
| Girl-girl |  | 19 | 0.02 | (0.38) | -0.28 | (0.54) | -0.38 | (0.63) |
| Girl-boy |  | 25 | 0.01 | (0.28) | 0.54 | (0.38) | 0.71 | (0.47) |
| Boy-girl |  | 16 | -0.43 | (0.35) | -0.07 | (0.55) | -0.40 | (0.62) |
| ***Grade effects*** |  |  |  |  |  |  |  |  |
| Low-low (ref.cat.) |  | 15 | -- | -- | -- | -- | -- | -- |
| High-high |  | 12 | 0.14 | (0.30) | -- | -- | 0.28 | (0.55) |
| Low-high |  | 13 | -0.61 | (0.42)^a^ | -- | -- | 1.68* | (0.71)^a^ |
| High-low |  | 12 | -0.62 | (0.38) | -- | -- | 0.63 | (0.66) |
| ***Age effects*** |  |  |  |  |  |  |  |  |
| Age-receiver |  | 26 | 0.02 | (0.08) | 0.02 | (0.11) | -0.02 | (0.15) |
| Difference in (relative) age |  | 26 | -0.01 | (0.06) | 0.02 | (0.08) | 0.01 | (0.11) |
| *Notes.* +*p* ≤ .10, **p* ≤ .05, ***p* ≤ .01, ****p* ≤ .001. ^a^Significant differences between classrooms. The parameter statistics ﻿of the network effects used in PNet are mentioned in parentheses (short names﻿). | | | | | | | | |

| **Appendix G*.*** ERGM meta-analysis results for lower-bound victimization networks in single-grade and multi-grade classrooms. | | | | | | | | |
| --- | --- | --- | --- | --- | --- | --- | --- | --- |
|  |  |  | ***Intercept (administrative multi-grade)*** | | ***Intercept +***  ***regular single-grade*** | | ***Intercept +***  ***pedagogical***  ***multi-grade*** | |
| Parameter | *Illustration* | *n* | *Est.* | *(S.E.)* | *Est.* | *(S.E.)* | *Est.* | *(S.E.)* |
| ***Network effects*** |  |  |  |  |  |  |  |  |
| Density (Arc) |  | 24 | -1.82 | (1.24)^a^ | -0.29 | (1.71)^a^ | -2.08 | (2.08)^a^ |
| Sinks (sink) |  | 23 | 3.23*** | (0.61)^a^ | -0.94 | (0.81)^a^ | -0.55 | (0.98)^a^ |
| Isolates (isolates) |  | 22 | 3.67*** | (0.92)^a^ | 0.26 | (1.23)^a^ | -0.82 | (1.58)^a^ |
| In-ties spread (AinS) |  | 19 | -0.08 | (0.72)^a^ | -0.69 | (1.05)^a^ | -0.03 | (1.25)^a^ |
| Multiple two-paths (A2P-T) |  | 15 | -0.05 | (0.20) | -0.11 | (0.27) | 0.21 | (0.32) |
| Shared in-ties (A2P-D) |  | 19 | -0.05 | (0.20) | 0.20 | (0.24) | 0.10 | (0.32) |
| ***Sex effects*** |  |  |  |  |  |  |  |  |
| Boy-boy (ref.cat.) |  | 24 | -- | -- | -- | -- | -- | -- |
| Girl-girl |  | 16 | 0.28 | (0.48) | -0.58 | (0.66) | -1.09 | (0.77) |
| Girl-boy |  | 22 | 0.32 | (0.59)^a^ | 0.41 | (0.83)^a^ | 0.25 | (1.05)^a^ |
| Boy-girl |  | 16 | -0.40 | (0.46) | -0.09 | (0.70) | -0.53 | (0.76) |
| ***Grade effects*** |  |  |  |  |  |  |  |  |
| Low-low (ref.cat.) |  | 14 | -- | -- | -- | -- | -- | -- |
| High-high |  | 10 | 0.08 | (0.37) | -- | -- | 0.38 | (0.60) |
| Low-high |  | 12 | -1.00* | (0.45) | -- | -- | 1.85* | (0.76) |
| High-low |  | 10 | -0.50 | (0.44) | -- | -- | 0.42 | (0.75) |
| ***Age effects*** |  |  |  |  |  |  |  |  |
| Age-receiver |  | 24 | 0.07 | (0.09) | -0.02 | (0.12) | -0.09 | (0.16) |
| Relative age-difference |  | 24 | 0.002 | (0.07) | 0.01 | (0.09) | -0.01 | (0.12) |
| *Notes.* +*p* ≤ .10, **p* ≤ .05, ***p* ≤ .01, ****p* ≤ .001. ^a^Significant differences between classrooms. Due to the lower-bound cut-off for victimization, two of the 26 classrooms were excluded because they had too few ties (one single-grade classroom had no ties and another pedagogical multi-grade classroom had only one tie). The parameter statistics ﻿of the network effects used in PNet are mentioned in parentheses (short names﻿). | | | | | | | | |

| **Appendix H*.*** ERGM meta-analysis results for victimization networks in single-grade and multi-grade classrooms – effects of sex-based age-difference. | | | | | | | | |
| --- | --- | --- | --- | --- | --- | --- | --- | --- |
|  |  |  | ***Intercept (administrative multi-grade)*** | | ***Intercept +***  ***regular single-grade*** | | ***Intercept +***  ***pedagogical***  ***multi-grade*** | |
| Parameter | *Illustratation* | *n* | *Est.* | *(S.E.)* | *Est.* | *(S.E.)* | *Est.* | *(S.E.)* |
| ***Network effects*** |  |  |  |  |  |  |  |  |
| Density (Arc) |  | 25 | -3.22*** | (0.78)^a^ | 1.33 | (1.07)^a^ | -1.09 | (1.24)^a^ |
| Sinks (sink) |  | 25 | 1.07* | (0.48) | 0.13 | (0.66) | 0.36 | (0.77) |
| Isolates (isolates) |  | 25 | 1.23+ | (0.68)^a^ | 1.52 | (0.95)^a^ | 0.13 | (1.07)^a^ |
| In-ties spread (AinS) |  | 23 | 0.75 | (0.54)^a^ | -0.96 | (0.76)^a^ | -0.11 | (0.93)^a^ |
| Multiple two-paths (A2P-T) |  | 20 | -0.02 | (0.16) | -0.14 | (0.22) | 0.18 | (0.30) |
| Shared in-ties (A2P-D) |  | 21 | 0.15 | (0.14) | -0.01 | (0.18) | -0.03 | (0.27) |
| ***Sex effects*** |  |  |  |  |  |  |  |  |
| Boy-boy (ref.cat.) |  | 25 | -- | -- | -- | -- | -- | -- |
| Girl-girl |  | 18 | 0.002 | (0.48)^a^ | -0.39 | (0.69)^a^ | -0.45 | (0.78)^a^ |
| Girl-boy |  | 24 | 0.24 | (2.68)^a^ | 0.16 | (3.79)^a^ | -5.90 | (4.23)^a^ |
| Boy-girl |  | 16 | -0.74 | (1.05)^a^ | -2.10 | (1.63)^a^ | -0.60 | (1.77)^a^ |
| ***Grade effects*** |  |  |  |  |  |  |  |  |
| Low-low (ref.cat.) |  | 15 | -- | -- | -- | -- | -- | -- |
| High-high |  | 12 | 0.17 | (0.32) | -- | -- | 0.28 | (0.59) |
| Low-high |  | 13 | -0.64 | (0.42)^a^ | -- | -- | 1.75* | (0.73)^a^ |
| High-low |  | 12 | -0.65 | (0.40) | -- | -- | 0.69 | (0.69) |
| ***Age effects*** |  |  |  |  |  |  |  |  |
| Age-receiver |  | 25 | 0.03 | (0.08) | 0.02 | (0.12) | -0.02 | (0.15) |
| Relative age-difference |  | 25 | -0.01 | (0.08) | 0.04 | (0.11) | 0.01 | (0.14) |
| ***Sex * age effects*** |  |  |  |  |  |  |  |  |
| Boy-boy age-diff. (ref.cat.) |  | 25 | -- | -- | -- | -- | -- | -- |
| Girl-girl age-diff |  | 18 | -0.03 | (0.11) | -0.005 | (0.18) | -0.01 | (0.20) |
| Girl-boy age-diff |  | 24 | 0.02 | (0.37)^a^ | -0.02 | (0.52)^a^ | 0.87 | (0.58)^a^ |
| Boy-girl age-diff |  | 16 | -0.06 | (0.12) | 0.0003 | (0.19) | 0.05 | (0.22) |
| *Notes.* +*p* ≤ .10, **p* ≤ .05, ***p* ≤ .01, ****p* ≤ .001. ^a^Significant differences between classrooms. One single-grade classroom was left out because the girl-girl age-diff effect estimate became unreasonably high. The parameter statistics ﻿of the network effects used in PNet are mentioned in parentheses (short names﻿). | | | | | | | | |
